# Ultracompact bottom-up photonic crystal lasers on silicon-on-insulator

**DOI:** 10.1038/s41598-017-10031-8

**Published:** 2017-08-25

**Authors:** Wook-Jae Lee, Hyunseok Kim, Jong-Bum You, Diana L. Huffaker

**Affiliations:** 1 School of Engineering, Cardiff University, Cardiff, CF24 3AA United Kingdom; 2 Department of Electrical Engineering, University of California Los Angeles, Los Angeles, California, 90095 United States; 3 Department of Electrical Engineering, Korea Advanced Institute of Science and Technology, Daejeon, 305-701 Republic of Korea; 4 School of Physics and Astronomy, Cardiff University, Cardiff, CF24 3AA United Kingdom; 5 California Nano-Systems Institute, University of California Los Angeles, Los Angeles, California, 90095 United States

## Abstract

Compact on-chip light sources lie at the heart of practical nanophotonic devices since chip-scale photonic circuits have been regarded as the next generation computing tools. In this work, we demonstrate room-temperature lasing in 7 × 7 InGaAs/InGaP core-shell nanopillar array photonic crystals with an ultracompact footprint of 2300 × 2300 nm^2^, which are monolithically grown on silicon-on-insulator substrates. A strong lateral confinement is achieved by a photonic band-edge mode, which is leading to a strong light-matter interaction in the 7 × 7 nanopillar array, and by choosing an appropriate thickness of a silicon-on-insulator layer the band-edge mode can be trapped vertically in the nanopillars. The nanopillar array band-edge lasers exhibit single-mode operation, where the mode frequency is sensitive to the diameter of the nanopillars. Our demonstration represents an important first step towards developing practical and monolithic III-V photonic components on a silicon platform.

## Introduction

Achieving strong confinement of light in subwavelength structures is a key feature for reducing the footprint and power consumption of on-chip light sources. III-V semiconductor nanowires or nanopillars have recently been investigated for potential ultracompact light sources in the field of micro and nanophotonics, because the nanowires can not only be grown on lattice-mismatched substrates such as silicon without buffer layers^1, 2^, but also allow strong confinement of photonic modes due to their large refractive index^3–7^. To date, however, most studies on nanowire-based lasers have been restricted to observing lasing from single nanowires with homogeneous bulk gain and multiple quantum wells (or dots) owing mainly to lack of control over the location, dimension, and orientation of the formed nanowires, which make single nanowire lasers unsuitable for on-chip and large-scale applications.

Photonic crystal (PhC) cavities are one of the most attractive candidates for the realization of compact and high-volume lasers due to their high quality (Q) factors and wavelength-scale mode volume. In particular, PhC membranes with air holes, which provide strong vertical confinement by total internal reflection^8^, have been extensively investigated with the aim of achieving on-chip integrated lasers^9–12^. However, further technological improvements are required to achieve large-scale III-V wafer bonding on silicon for high-volume manufacturing. As an alternative approach, monolithic nanopillar (or nanowire) PhC structures grown by selective-area epitaxy would be of great interest from the point of view of practical applications, because III-V semiconductor nanopillars with atomic-scale sidewall ({1–10} family of planes) roughness^13^ offer low optical losses and easy carrier injection^14, 15^. Furthermore, these nanopillar PhC structures can be directly integrated on 3D structured silicon-on-insulator (SOI) substrates^16, 17^, where SOI is a promising platform for photonic integrated circuits. These nanopillar PhCs allow strong interactions between resonant modes and surrounding environments, suggesting high possibilities for the application to lab-on-a-chip devices. However, achieving vertical confinement has been a challenge in these pillar-type PhC cavities, because a large refractive index difference between nanopillars (or rods) and growth substrates is required to prevent leakage into the substrates. Several methods have been reported to satisfy this requirement and demonstrate lasing, such as top-down etched microrod array PhCs with multiple quantum wells bonded on a low-index substrate^18^, bottom-up nanopillar PhCs with III-V heterostructures detached from the growth substrate^19^, and selectively wet-etched rod PhCs with multiple quantum wells on the growth substrate^20^. Therefore, the dilemma lies in the fact that nanopillar-based PhC structures can be grown directly on lattice-mismatched substrates like silicon but the Q factor of such PhCs can be significantly degraded by weak vertical mode confinement because of the small refractive index contrast.

We demonstrate in this study room-temperature lasing in InGaAs nanopillar PhCs directly grown on SOI by optical pumping. Catalyst-free selective-area epitaxy (SAE) by metal-organic chemical vapor deposition (MOCVD) is employed to grow nanopillar PhC cavities on pre-defined periodic nanoholes in a dielectric mask. As it is well-known that nanohole geometries (e.g., pitch and diameter) affect growth rates^19, 21^ and material compositions^22^ in the SAE approach, we concentrate on defect-free PhC cavities with a constant pitch and nanohole diameter to ensure good structural and compositional uniformity in nanopillars. Strong horizontal confinement enhanced by slow-light in the 7 × 7 nanopillar array is achieved through the uniform growth of the nanopillars with atomic-scale sidewall roughness and *in-situ* surface passivation. Utilizing an SOI layer with an appropriate thickness enables the slow light mode to be trapped inside the nanopillars. We also show that lasing wavelengths of the nanopillar PhC lasers can be widely tuned from 1020 nm to 1300 nm by changing the pitch and the diameter.

## Results and Discussion

**Figure 1:**
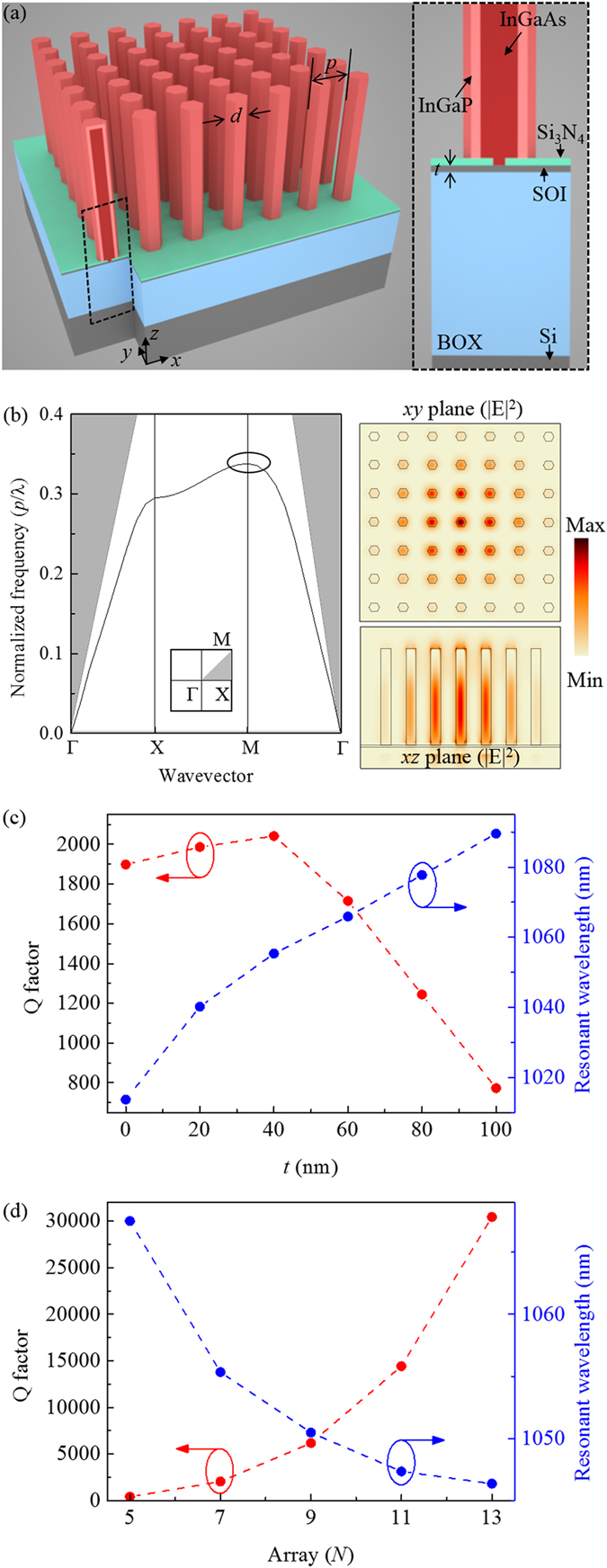
(**a**) Schematic illustrations of the 7 × 7 nanopillar PhC laser with InGaAs/InGaP core-shell structures on an SOI substrate with an SOI layer thickness of *t*. *d* and *p* indicate the diameter and the pitch of nanopillars, respectively. (**b**) Corresponding TM mode photonic band structure of the square lattice nanopillar array (left). The grey area denotes the region above the light line in air. |E|^2^ field profiles of the first band-edge mode (black circle) calculated by the FDTD method are also shown (right). Calculated Q factors (filled red circles) and resonant wavelengths (filled blue circles) as a function of *t* in 7 × 7 nanopillar arrays (**c**) and a function of an array size at *t* = 40 nm (**d**).

Figure 1(a) shows schematic illustrations of the nanopillar PhC laser, which is comprised of bottom-up InGaAs/InGaP core-shell structures on an SOI substrate. The InGaP shell is employed to reduce non-radiative surface recombination by passivating the surface of InGaAs nanopillars. The corresponding photonic band structure of the first TM mode in a three-dimensional nanopillar array with 350 nm pitch, 130 nm diameter and 800 nm height is plotted using the finite-difference time-domain (FDTD) method, as seen in Fig. 1(b). We focused on the first TM mode near the band-edge (M-point, black circle) below the light line. The electric field intensity profiles (|E|^2^) of the first M-point band-edge mode are also shown in the same figure, clearly exhibiting well-confined field in the 7 × 7 nanopillar array. In simulations, the refractive indices of InGaAs/InGaP nanopillars and Si are assumed to be 3.4 and 3.55, respectively. Using the FDTD method, the Q factors and resonant wavelengths are obtained with various thicknesses (*t*) of the SOI layer and the number of nanopillars constituting the array (*N* × *N*), as shown in Fig. 1(c and d), respectively. By calculating the Q factor versus the thickness of the SOI layer, it was found that the presence of the SOI layer (40-nm-thick in this case) leads to the highest Q factor, which means that an optical buffer layer is required to prevent the leakage into a buried oxide (BOX) layer (see Supplementary Fig. S1). The Q factor can also be increased as the number of nanopillars increases due to enhanced lateral confinement. In this study, we employed a 7 × 7 nanopillar array on a 40-nm-thick SOI layer, which has an ultracompact cavity volume of 2300 × 2300 × 800 nm^3^ and the Q factor of 2042.

**Figure 2:**
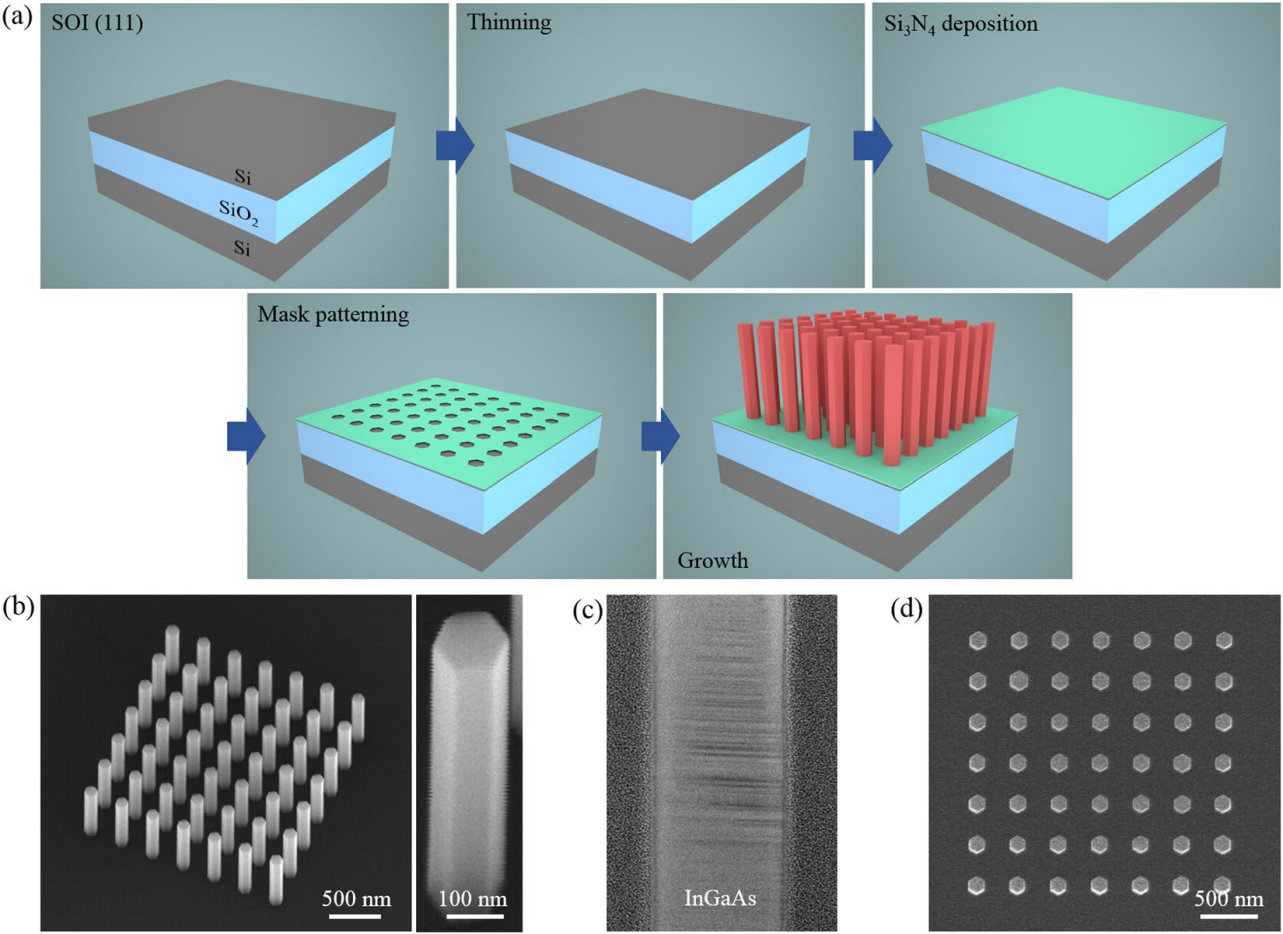
(**a**) Schematic diagram of nanopillar PhCs fabrication process. (**b**) 30° tilted and enlarged SEM images of as-grown InGaAs/InGaP core-shell nanopillars with *d* = 130 nm, *p* = 350 nm, and a height of 800 nm on the 40-nm-thick SOI substrate. (**c**) Cross-sectional TEM image of an InGaAs/InGaP core-shell nanopillar. (**d**) Top-view SEM image showing uniformly grown nanopillar array.

The fabrication processes are illustrated in Fig. 2(a). A 30° tilted and magnified scanning electronic microscopy (SEM) images of as-grown InGaAs/InGaP core-shell nanopillar PhCs are displayed in Fig. 2(b). The nanopillar in the magnified SEM image exhibits a smooth surface morphology with a diameter (*d*) of 130 nm and a height of 800 nm. As shown in the cross-sectional TEM image in Fig. 2(c), the nanopillars are free of threading dislocations, despite the lattice mismatch of ~5% between InGaAs and silicon. However, high density of stacking defects is observed, because crystal structures are typically difficult to control in the SAE growth. The top-view SEM image in Fig. 2(d) shows that the nanopillars with uniform diameters are vertically grown on the SOI layer. The effect of InGaP shell passivation is reported elsewhere^23^.

**Figure 3:**
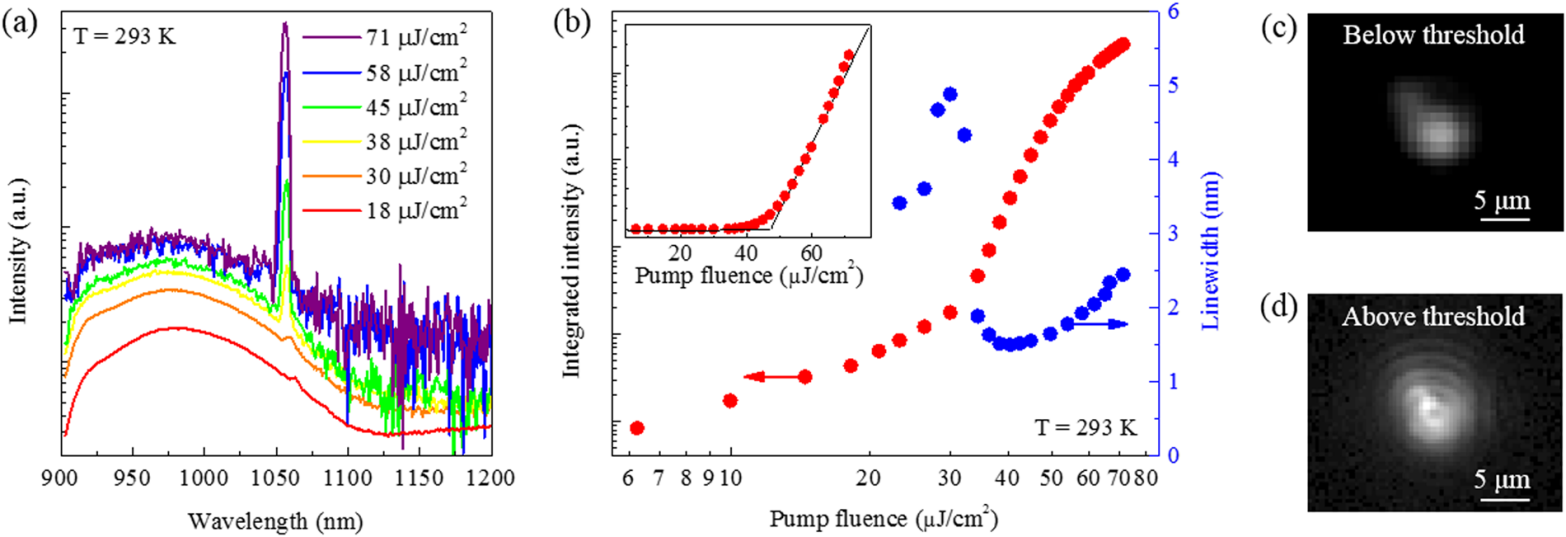
(**a**) Room-temperature emission spectra at various pump powers. The laser peak at a wavelength of 1057 nm steeply increases above threshold. (**b**) L–L curve of the nanopillar array band-edge laser with a threshold of ~45 µJ/cm^2^ (filled red circles) and corresponding spectral linewidth (filled blue circles). Integrated intensities are obtained by Gaussian fitting of the spectra. Inset: L–L curve on a linear scale. Optical images of the laser emission below threshold (**c**) and above threshold (**d**). Interference fringe patterns are captured above threshold.

The microphotoluminescence (µ-PL) spectra for the nanopillar PhCs measured at room-temperature under various pump powers are plotted in Fig. 3(a). A pulsed supercontinuum laser (NKT Photonics, SuperK EXTREME) was utilized to pump the nanopillar array (*λ*_pump_ =660 nm, pulse length 30 ps, repetition rate 1.95 MHz). The pump light was focused normal to the nanopillar array with a diameter of 1.8 µm through an objective (Mitutoyo, M Plan Apo NIR, × 50/0.42 numerical aperture), and the emitted light was collected by the same objective. The collected light was analyzed by a spectrometer and a liquid nitrogen-cooled 2D focal plane array InGaAs detector (Princeton instruments SP-2500i and 2D-OMA). The pump light was blocked by filters for these measurements. Broad spontaneous emissions are seen at low pump powers, while the band-edge cavity mode becomes dominant by increasing the pump power at a wavelength of 1057 nm, where the cavity resonant wavelength agrees well with the theoretical calculation using the FDTD method. It is observed that the cavity mode intensity is more than one order of magnitude larger than the spontaneous emission for the pump fluences above 58 µJ/cm^2^. We note that the nanopillar PhC laser shows single-mode operation at room-temperature, which is crucial for optical sensing and communication applications. Figure 3(b) shows the integrated intensity of the cavity mode as a function of pump power (light-in light-out (L–L) curve) on a log-log scale, plotted together with the spectral linewidth. The measured integrated intensities manifest lasing action such as an S-shaped behavior and a sudden decrease of the linewidth around threshold. The lasing threshold is estimated to be ~45 μJ/cm^2^ from these characteristics, which is also substantiated from the position of the kink between two linear regions in the inset of Fig. 3(b). The measured linewidth at the threshold is ~1.5 nm, equivalent to a Q factor of ~704. Figure 3(c and d) show optical images of the nanopillar PhC laser below and above threshold. Strong coherent light emission with interference fringe patterns is observed above threshold, while such interference fringes are not captured below threshold. We obtained a donut-shaped lasing emission from the nanopillar PhC laser, where the laser emission was azimuthally polarized (see Supplementary Fig. S2). The discrepancy between the experimental and simulated Q factors may be explained by slight differences in the geometry of nanopillars such as height and diameter. We have also measured the photoluminescence spectra of a nanopillar array on a 220-nm-thick SOI layer (see Supplementary Fig. S3). It is confirmed that no resonance peak is observed at pump powers above threshold due to the leakage into the SOI layer.

**Figure 4:**
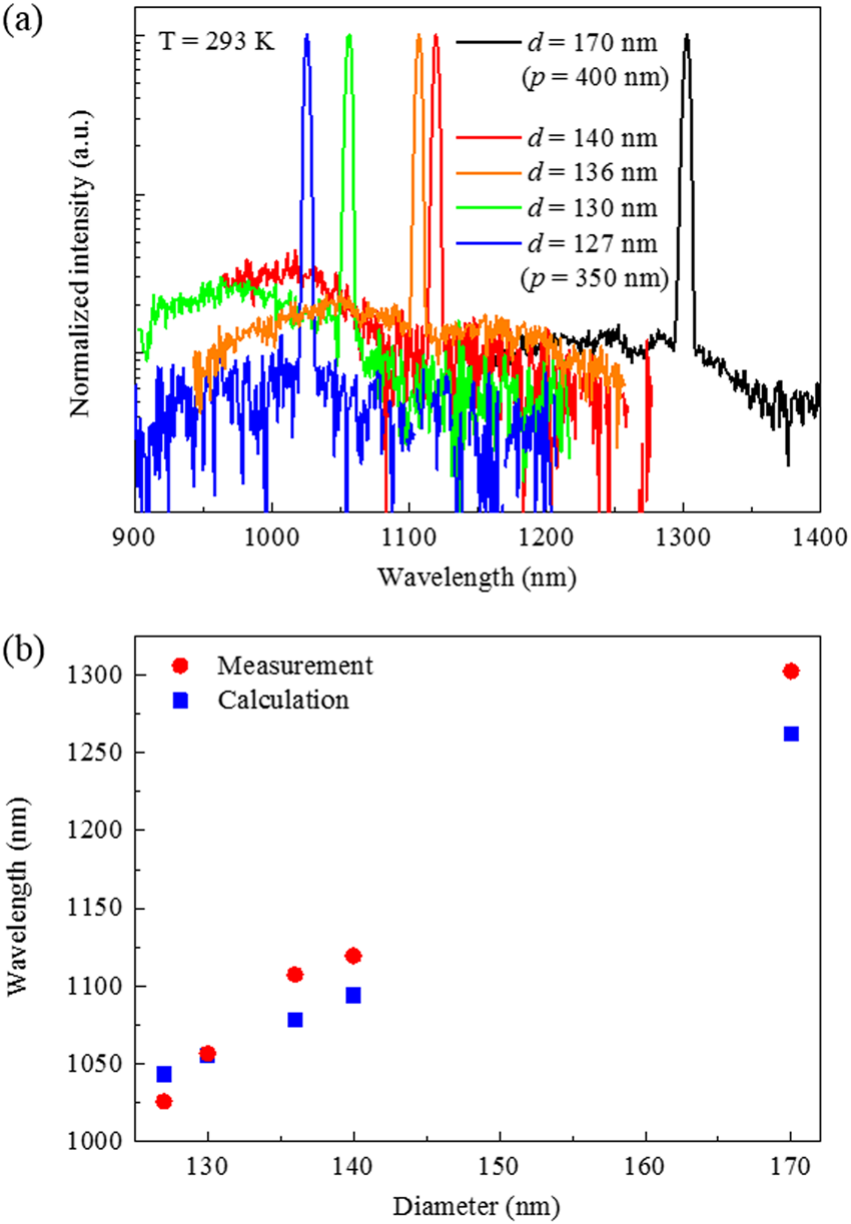
(**a**) Diameter-dependent emission spectra of nanopillar PhC lasers measured at room-temperature plotted in a log scale. Lasing up to a telecommunication wavelength of 1300 nm (*d* = 170 nm and *p* = 400 nm) is obtained. (**b**) Measured lasing wavelengths and calculated resonant wavelengths of the first TM modes (M-point).

It should be stressed that the nanopillar PhC lasers show diameter dependency in their lasing wavelengths, which suggests a potential application to nanopillar PhC sensors with direct real-time detection of molecules binding to the nanopillars (see Supplementary Fig. S4). As shown in Fig. 4(a), the diameter variation of only 13 nm results in the lasing wavelength shift of 93 nm at the fixed pitch of 350 nm, revealing a strong dependence of lasing wavelengths on the nanopillar diameter (see Supplementary Fig. S5). By changing the pitch to 400 nm and increasing the indium composition in InGaAs nanopillars, we also demonstrated the nanopillar PhC laser on a silicon platform operating at the telecommunications wavelength of 1300 nm, which will be useful in silicon photonics as an on-chip laser. Additionally, the measured lasing wavelengths are compared with the calculated resonant wavelengths of the first M-point band-edge modes. The refractive indices of nanopillars and Si were fixed at 3.4 and 3.55, respectively. Figure 4(b) reveals good agreement between the measured data and calculations, and this result supports that the observed lasing emission occurred at the first M-point band-edge mode. The small discrepancy between the experimental and calculated values can be attributed to the diameter difference of the individual nanopillars in an array and the refractive index change with respect to the material composition at a given wavelength. It is worth mentioning that the diameter of nanopillars can be lithographically controlled by changing the diameter of nanoholes. Since epitaxy conditions such as the growth temperature, V/III flow rate ratio and total flow rates also affect the aspect ratio (height/diameter) and the total volume of nanopillars^24, 25^, the diameter and height of nanopillars can be individually controlled by combining these lithographic and epitaxial approaches. This suggests that the proposed bottom-up PhC lasers exhibit a high degree of freedom in tuning the lasing wavelengths.

Recently, we have theoretically investigated that the M-point band-edge mode in nanopillar arrays can be efficiently coupled into waveguides while maintaining a high Q factor^17^. It has been shown that the mode below the light line is vertically confined in nanopillars, and well-guided to the waveguides with a similar refractive index to that of the nanopillars. Thus, the nanopillar PhC laser monolithically integrated on the silicon platform and operating at room-temperature will be of great interest and benefit to the researchers in the active field of sensing and photonics. In addition, as two-dimensional band-edge lasers are attractive and suitable for nonlinear optics, laser-based surgery, and military applications because of their high output power^26, 27^, our demonstration provides an approach to scale down high-power lasers on the silicon platform.

We have demonstrated ultracompact PhC lasers, which consist of 7 × 7 nanopillar arrays, operating at room-temperature under optical pumping. The SAE method was adopted to grow uniformly well-ordered nanopillar arrays, and an optimized thickness of SOI layer was employed to achieve strong vertical confinement. Diameter dependence of the lasing characteristics indicates a new approach to integrated nanosensors on a silicon platform, which can potentially enable cost-effective lab-on-a-chip biosensing. Using a bottom-up approach allows to form PhC lasers towards wafer-scale integration directly on SOI substrates, implying that our concept of nanopillar PhCs provides an additional degree of freedom in future photonic devices.

## Methods

### Fabrication

We used a lightly p-doped (Boron, 10 Ω.cm) SOI (111) wafer with a 2000-nm-thick BOX layer as a growth substrate. The SOI layer was thinned to 40 nm by a combination of thermal oxidation and oxide removal by a 6:1 buffered oxide etch. A mask layer (20-nm-thick Si_3_N_4_) was deposited on the SOI layer and nanohole arrays with 60-nm-wide nanoholes were defined by electron-beam lithography and dry etching.

### Growth

Nanopillar growth was carried out in a low-pressure (60 Torr) vertical MOCVD reactor (Emcore D-75). The reactor temperature was first ramped up to 850 °C and held for 10 min for thermal de-oxidation. The temperature was then ramped down to 680 °C, and a short GaAs segment was grown on exposed nanoholes for 3 min by flowing triethylgallium (TEGa) and *tert*-butylarsine (TBA). The partial pressure of TEGa and TBA was 2.25 × 10^−5^ atm and 1.36 × 10^−3^ atm, respectively. InGaAs nanopillars were grown on top of GaAs segments at the same temperature for 11 min, under the partial pressure of trimethylindium (TMIn) = 8.70 × 10^-6^ atm, TEGa = 2.06 × 10^−5^ atm, and TBA = 1.36 × 10^−3^ atm, which corresponds to the gas phase indium composition of 29% and V/III flow rate ratio of 47. Finally, thin InGaP shells were grown for *in-situ* surface passivation at 600 °C for 45 s, under the partial pressure of TMIn = 9.27 × 10^−6^ atm, TEGa = 2.81 × 10^−6^ atm, and *tert*-butylphosphine (TBP) = 1.62 × 10^−3^ atm. After the growth, the reactor was cooled down to 300 °C by flowing TBP to prevent desorption of InGaP shells.

## Electronic supplementary material

Supplementary information
