# Supplementary material for: Ultracompact bottom-up photonic crystal lasers on silicon-on-insulator
Source: Sci Rep. 2017 Aug 25;7:9543. doi: 10.1038/s41598-017-10031-8 (PMC5573312; doi:10.1038/s41598-017-10031-8)
Supplement: Supplementary file 1 — Supplementary information [file 41598_2017_10031_MOESM1_ESM.doc]

**Supplementary Information**

**Ultracompact bottom-up photonic crystal lasers on silicon-on-insulator**

**Wook-Jae Lee**^1,‖,*^, **Hyunseok Kim**^2,‖,*^, **Jong-Bum You**^3^, and **Diana. L. Huffaker**^2,4,5^

^1^School of Engineering, Cardiff University, Cardiff CF24 3AA, United Kingdom

^2^Department of Electrical Engineering, University of California Los Angeles, Los Angeles, California 90095, United States

^3^Department of Electrical Engineering, Korea Advanced Institute of Science and Technology, Daejeon 305-701, Republic of Korea

^4^School of Physics and Astronomy, Cardiff University, Cardiff CF24 3AA, United Kingdom

^5^California Nano-Systems Institute, University of California Los Angeles, Los Angeles, California 90095, United States

**Figure S1.** Calculated lateral (Q_lateral_) and vertical (Q_vertical_) Q factors as a function of the SOI layer thickness (*t*) in 7 × 7 nanopillar arrays. The vertical Q factor shows a maximum value at *t* = 40 nm while the lateral Q factor decreases with increasing *t* due to the lateral leakage into the SOI layer.

**Figure S2.** Measured angle-resolved lasing mode images as a function of the polarization angle. Left: horizontal polarization. Center: vertical polarization. Right: 45˚ tilted polarization. The lasing mode images clearly show the angle-dependence because the laser emission is azimuthally polarized.

**Figure S3.** Room-temperature photoluminescence spectra of an InGaAs/InGaP core-shell nanopillar array on a 220 nm-thick SOI substrate at above-threshold pump power. No lasing (cavity mode) is observed even at 4 times threshold.

**Figure S4.** Resonant wavelength change (*∆λ_res_*) as a function of the thickness (*t_cladding_*) and refractive index (*n_cladding_*) of the cladding layer. Despite the lower refractive index of the thin cladding layer than the nanopillar, the nanopillar PhC lasers represent the diameter dependence of the resonant wavelength, which offers potential means for monitoring environmental change.

**Figure S5.** Top-view SEM images of as-grown InGaAs/InGaP core-shell nanopillar arrays on SOI substrates with various diameters (*d*) and pitches (*p*) under the same magnification.
